# Spontaneous knot formation complication of double J: two case reports

**DOI:** 10.1186/s13256-024-04395-5

**Published:** 2024-03-13

**Authors:** Ahmet Can Kolu, Serkan Akan

**Affiliations:** Department of Urology, University of Health Sciences, Fatih Sultan Mehmet Research & Training Hospital, 34758 Istanbul, Turkey

**Keywords:** Knotted ureteral stent, Urological complication, Ureteral stent

## Abstract

**Background:**

Use of ureteral stents has become an integral part of urological practice. However, it also brought with it many complications. Double J (DJ) stent knotting is a rare stent complication, and only a few cases have been reported in the literature. Although the exact cause is unknown and, in the literature, it is generally thought that knots occur due to traction. In this case report we present for the first time that spontaneous knots can occur due to ureteral peristalsis or ureteral anomalies.

**Case presentation:**

Two patients (67 and 35 aged-Caucasian person) with ureteral stones who presented to the emergency department with colicky pain and had no previous history of urological surgery. We observed knot formation in the routine urinary system radiographs taken before stent removal in two patients whose ureters were observed to be narrow during endoscopic ureteral stone treatment. The stents were successfully removed using gentle traction under general anesthesia.

**Conclusions:**

We discussed the cause and solution of spontaneous knot formation. We emphasized the importance of the direct urinary system radiograph taken before DJ stent removal.

## Introduction

The ureteral stents, which become an integral part of modern urological practice, provide urinary tract access, dilation of urinary strictures, removal of kidney stones, and temporary drainage of urine. There are various size (14–32 cm), diameter (3–8 F), hardness, body, tip shapes (pigtail, double pigtail-JJ) and coating (carbon, hydrophilic, heparin) ingredients for the purpose of use.

During their time with ureteral stents, 80% of patients report experiencing some degree of discomfort. Additionally, the use of ureteral stents may lead to serious complications such as stent migration and stent encrustation. Although spontaneous knotting of the ureteral stent has been reported before, it is one of the rare complications in the literature. There are a variety of techniques reported for the removal of a knotted double J (DJ) stent, from simple traction to open surgery.

## Case report

The first case, a 67-year-old man (Caucasian), applied to our clinic with a complaint of left side pain that had been going on for a month. On physical examination, positive left costovertebral angle (CVA) tenderness was observed (Table 1). He had hypertension and diabetes. There was a stone disease in his father. The patient had no previous history of renal colic, urinary system stone disease or any surgical intervention. In the imaging performed for the patient, “Grade 2 ectasia in the upper collecting system of the left kidney, double ureter appearance on the left and suspicious stone image at the level of the left iliac crossover” was observed; ureterorenoscopy (URS) was planned for the patient. During cystoscopy, a close monitoring of the patient’s left ureteral orifice revealed an attempt to insert a sensor guide. However, it failed to transition from the middle ureter to the proximity. Then, the distal ureter was entered through the left ureteral orifice by applying ureteral balloon dilation under the guidance of a sensor guide. The ureter at the iliac cross level was passed with difficulty, but due to stenosis, it could not be advanced further proximally; 4.8 Fr 26 cm DJ was placed and it was seen that both ends were bent under fluoroscopy.

**Table 1:** Timeline

Time (t)	t_0_	t_1_	t_2_	t_3_
Important dates	Initial presentation	After 2 weeks (URS time)	1 day after URS	6 weeks after URS

Time intervals are the same for both patients

*URS* Ureterorenoscopy

**Fig. 1:**
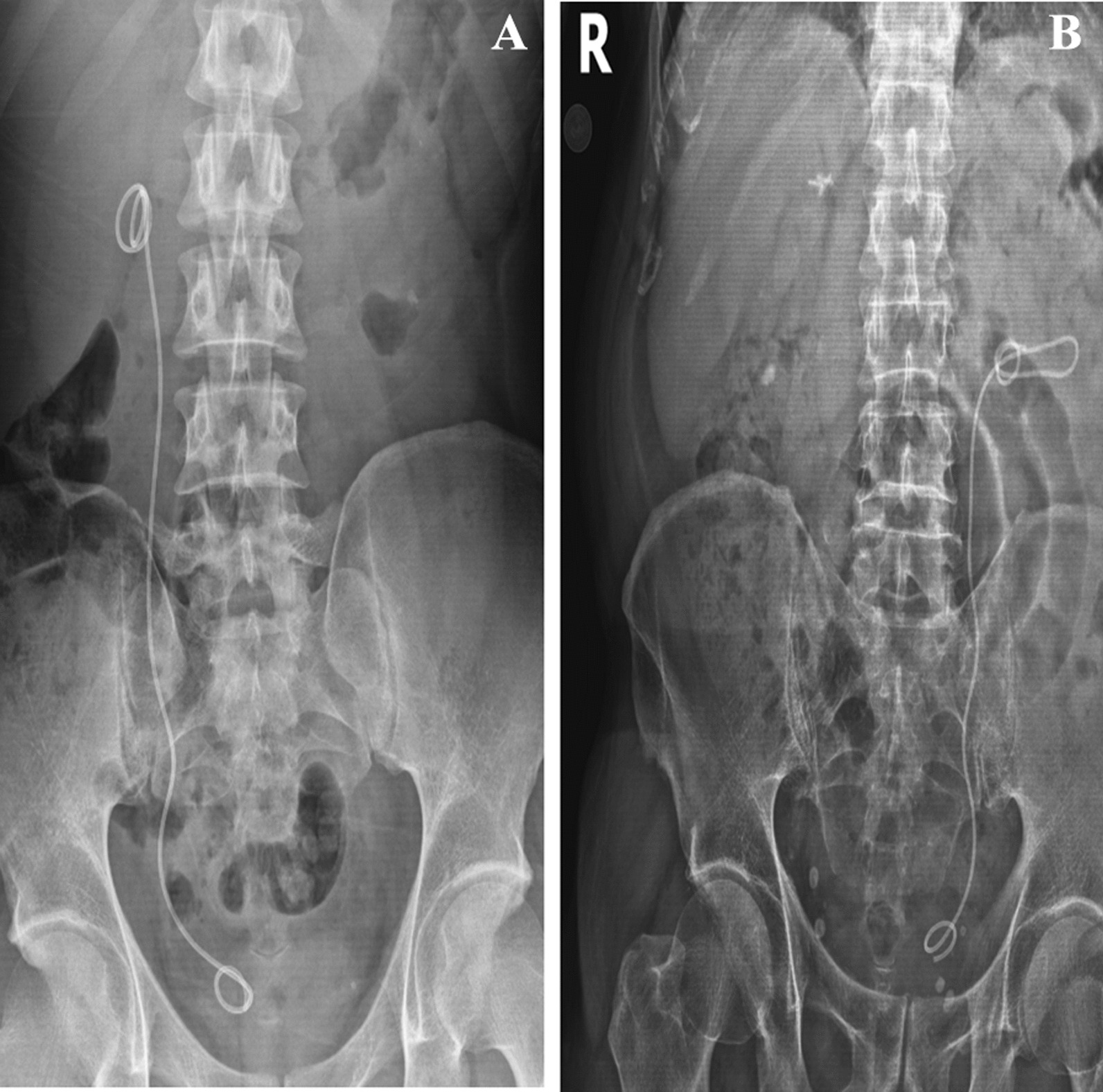
**A** Case 1 direct urinary system graphic after Ureterorenoscopy. **B** Case 2 direct urinary system graphic after Ureterorenoscopy

The second case (Caucasian), aged 35, had complaints of right-side pain for four months. On physical examination, positive right CVA tenderness was observed (Table 1). The patient had no comorbidity. There was a stone disease in his family. A calculus of 3–4 mm in size was observed in the right distal ureter in the imaging performed on the patient, who had a previous history of passing spontaneous urinary system stones but did not have a previous surgical history. During the URS procedure performed 2 weeks later, annular stenosis was observed 2 cm ahead of the right orifice and was corrected with balloon dilatation. Endoscopic ureteral stone treatment was performed with holmium laser. At the end of the surgery, a 4.8 Fr 26 cm DJ was placed into the right ureter and the procedure was terminated. No perioperative or postoperative complications were observed in both cases, and the urinary system was visualized on the radiography on the first day after the operation (Fig. 1A, B).

**Fig. 2:**
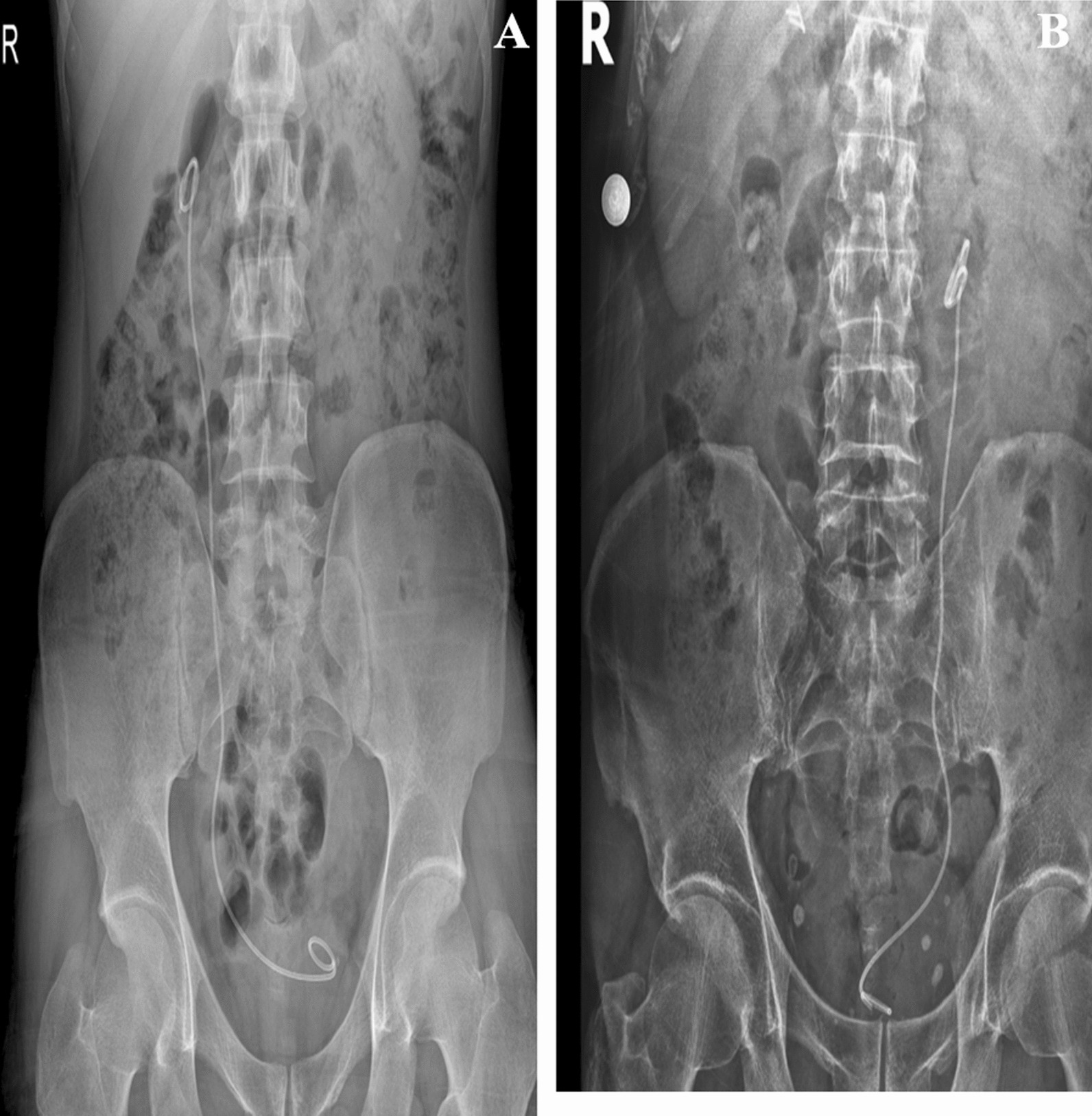
**A** Case 1 direct urinary system graphic taken before Double J removal. **B** Case 2 direct urinary system graphic taken before Double J removal

**Fig. 3:**
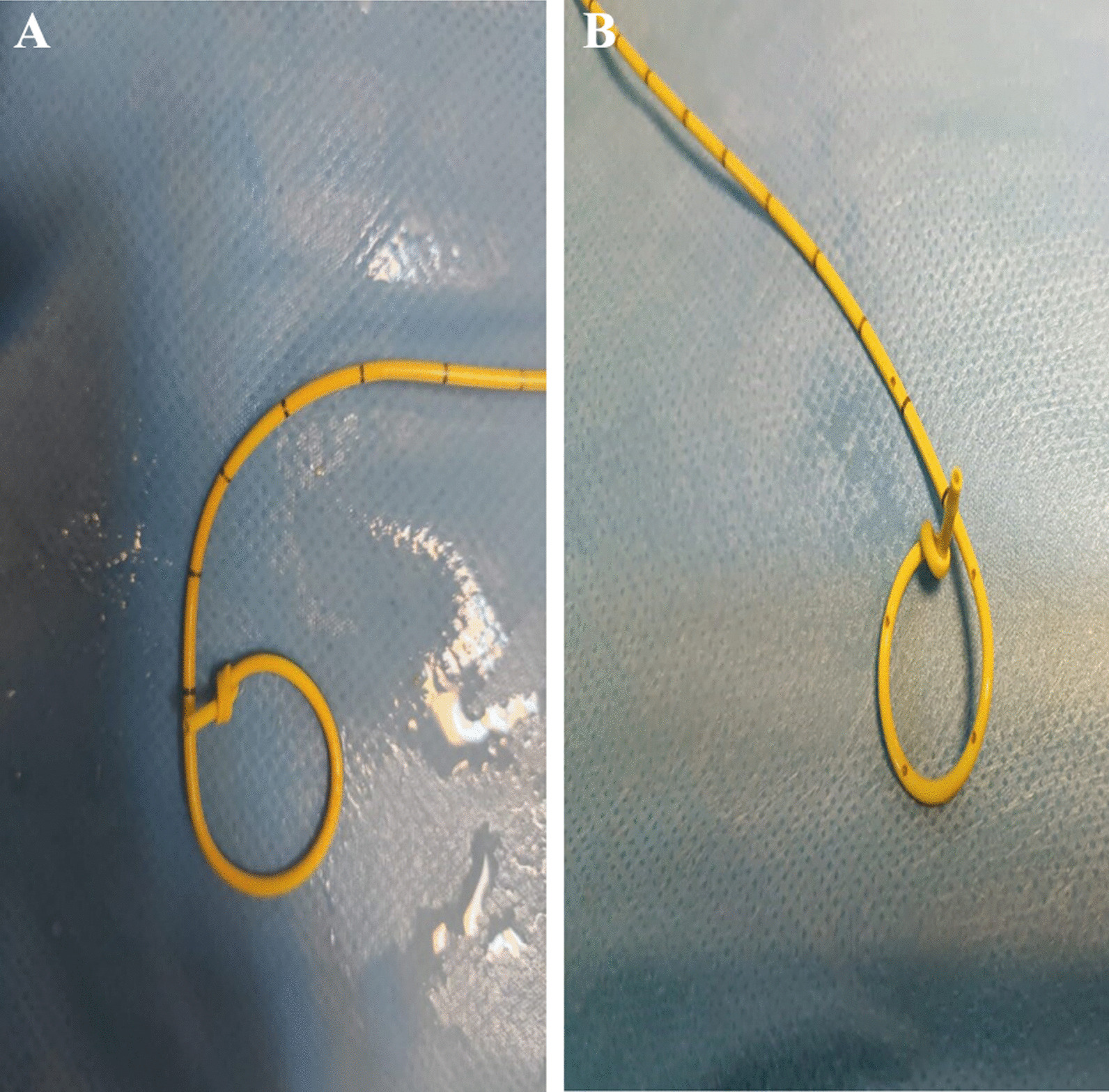
**A** Case 1 Double J ureteral stent with knot formation at proximal end. **B** Case 2 Double J ureteral stent with knot formation at proximal end

In both cases, when the urinary system radiographs were repeated six weeks later for routine DJ stent removal; spontaneous knotting was observed at the proximal end of the DJ stents (Fig. 2A, B). Both of patients no bothersome symptoms were reported, and the physical examination was strictly normal. Considering the possibility that the procedure could be complicated, DJ stent removal was planned under general anesthesia. After the distal coil of the DJ stents was corrected, gentle traction was applied toward the contralateral bladder wall. DJ stents were barely removed without complications, and proximal ends were found to be coming in knotted (Fig. 3A, B). A 7Fr ureteral catheter was placed over the guide wire to the renal pelvis. The ureteral catheter was removed on postoperative day 1; After observation, the patient was discharged with nonsteroidal anti-inflammatory drug (NSAII) and antibiotic treatment.

## Discussion

Ureteral DJ stent complications include irritative micturition symptoms, suprapubic pain, costovertebral pain, vesicorenal reflux, stent malposition, hematuria, urinary tract infection, fever, encrustation, stent migration, stent rupture, ureteral perforation, erosion and fistulization. To our best knowledge, only a few cases have been identified in the literature since Groeneveld et al. first reported it in 1989 [1]. Table 2 summarized the previously recent published cases regarding DJ stent knotting. Knotted formation is a rare complication, with only 40 cases being described and can be vexing to manage. In the vast majority of reported cases (92.5%), knotting was observed at the proximal end of the DJ stent. In our patients and in the majority of other cases of knotting, the patients were asymptomatic and the cases typically presented with unexpected resistance during DJ stent removal.

**Table 2:** Literature review of knot formation

Case	Year	Author	Age/Gender	Location	Technique of removal
1	1989	Groeneveld et al. [1]	NA	Proximal	Gentle traction
2	1990	Das et al. [9]	45/M	Distal	Gentle traction
3	1992	Braslis et al. [10]	37/F	Proximal	Percutaneous nephrostomy (PCN) removal
4	1994	Kundagi et al. [11]	53/M	Proximal	PCN removal
5	1995	Flam et al. [7]	86/M	Proximal	2nd DJ stent and URS
6	1998	Baldwinn et al. [6]	73/M	Proximal	Guidewire (Superstiff) to untie the knot
7	2002	Quek M et al. [12]	66/F	Mid	Gentle traction
8	2005	Sighinolfi et al. [4]	48/M	Proximal	Continuous traction for 3 days and ESWL
9	2005	Kondo et al. [13]	37/M	Proximal	Ureterotomy
10	2006	Eisner et al. [5]	82/F	Proximal	Gentle traction (Valsalva)
11	2007	Basavaraj et al. [14]	70/F	Proximal	PCN and gentle traction
12	2009	Rivalta et al. [15]	83/M	Proximal	Gentle traction with vaseline lubrication
13	2010	Picozzi et al. [16]	41/F	Proximal	Gentle traction
14	2011	Tempest et al. [17]	NA	Proximal	URS and Holmium laser
15	2011	Richards et al. [18]	67/M	Proximal	URS and Holmium laser
16	2012	Moufid et al. [19]	32/M	Proximal	2nd DJ stent and gentle traction
17	2012	Karaguzel et al. [20]	53/M	Proximal	URS and gentle traction
18	2012	Nettle et al. [21]	43/M	Proximal	URS and Holmium laser
19	2012	Bhirud et al. [22]	41/M	Proximal	Percutaneous removal with 26F nephroscope
20	2015	Ahmadi et al. [8]	45/M	Proximal	URS and Holmium laser
21	2015	Ahmadi et al	43/M	Proximal	URS and Holmium laser
22	2015	Ahmadi et al	71/M	Proximal	URS and percutaneous retrieval at later date
23	2015	Ahmadi et al	55/M	Proximal	URS and Holmium laser
24	2015	Kim et al. [23]	53/M	Proximal	Percutaneous and Terumo Guidewire
25	2015	Manohar et al. [24]	65/M	Proximal	Staged percutaneous antegrade removal
26	2015	Manohar et al.	65/F	Proximal	URS and Holmium laser
27	2015	Manohar et al.	55/F	Proximal	URS and Holmium laser
28	2015	Manohar et al.	59/M	Proximal	Gentle traction
29	2020	Bradshaw et al. [3]	57/F	Proximal	URS and dilation
30	2020	Cho et al. [25]	62/M	Proximal	URS and guidewire
31	2021	Choo ZW et al. [26]	73/M	Proximal	URS and Holmium laser
32	2022	Agarwal et al. [27]	77/M	Proximal	Access sheath assembly
33	2022	Agarwal et al	44/M	Proximal	Access sheath assembly
34	2022	Agarwal et al	65/M	Proximal	Access sheath assembly
35	2022	Gur et al. [28]	25/F	Mid	Guidewire
36	2022	Jendouzi et al. [2]	20/M	Proximal	URS and Holmium laser
37	2022	Divya et al. [29]	5/M	Proximal	Percutaneous and cystoscopically
38	2023	Weeratunga et al. [30]	73/M	Proximal	Loop-snare technique
39	2023	Present study	67/M	Proximal	Gentle traction
40	2023	Present study	35/M	Proximal	Gentle traction

*DJ* double J, *ESWL* extracorporeal shock wave lithotripsy, *PCN* percutaneous nephrostomy, *URS* Ureterorenoscopy

In the majority of reported cases, no abnormal appearance was detected in the urinary system radiograph taken before stent removal [2], which would suggest knotting in the DJ stent; it has been stated that the knot may form due to traction during extraction. However, in our cases, knot formation was observed to develop spontaneously immediately after URS, without any intervention or traction. This suggests that knot formation may develop due to the ureter’s own peristalsis or secondary to balloon dilatation applied to the abnormal ureter.

In approximately one-third of reported cases, the DJ stent could be removed with gentle traction and the condition was treated successfully [3]. However, this procedure carries risks for these patients as it may make the existing knot tighter and increase the degree of complications. If strong resistance is encountered during DJ stent removal, alternative interventions should be considered to avoid causing serious ureteral trauma or loss of renal function [3]. In previous years, “the use of sterile Vaseline in addition to traction” has been tried; There are suggestions such as “securing the distal end of the DJ catheter to the leg with a catheter band and providing continuous traction for 3 days” or “applying extracorporeal shock wave lithotripsy (ESWL) to the migrated area of the knotted stent” [4].

In another case where knot formation was observed twice in the same patient, no additional intervention was required to open the second knot formation; spontaneous resolution of the node has been associated with the Valsalva effect achieved by recurrent severe coughs [5]. Valsalva has been suggested as an easy and harmless treatment before invasive procedures for removing knotted stents.

Baldwin et al. used an “Amplatz 0.038 super stiff guidewire” at the proximal end of the stent to solve knot formation [6]. Flam et al. placed a second ureteral stent next to the knotted stent, and a week later, the stent was removed with 5Fr alligator forceps [7]. Endourologically, breaking down the knot formation with a holmium laser and removing the stent has been suggested in the literature as another method [8]. Removal of knot-forming stents via percutaneous or open surgery should only be performed after failure with other techniques. Urologists should be aware of the possibility of knot formation in the stent if difficulty is encountered during stent removal.

## Conclusion

Although the literature shows that a knot can occur with traction during DJ stent removal, we also believe that a spontaneous node may be caused by ureteral peristalsis or ureteral anomalies. Therefore, the routine use of a urinary system graph (X) for all patients before the release of the DJ stent can prevent potential complications due to spontaneous knot formation.

Even if we do not see knot formation on the x-ray, in all cases with ureteral stent in which difficulty is experienced during removal, the possibility of stent knotting should always be kept in mind and therapy planned accordingly.

## Data Availability

Not applicable.

## Abbreviations

CVA: Costovertebral angle
DJ: Double J
ESWL: Extracorporeal shock wave lithotripsy
NSAII: Nonsteroidal anti-inflammatory drug
PCN: Percutaneous nephrostomy
URS: Ureterorenoscopy
